# Assessing the Impact of a University Transition Online Course on Student Continuation Using Statistical Matching Methods

**DOI:** 10.1177/0193841X251339686

**Published:** 2025-05-02

**Authors:** Billy Wong, Lydia Fletcher

**Affiliations:** 1 Institute of Education, 6816 University of Reading, Reading, UK; 2 Centre for Quality Support and Development, 6816 University of Reading, Reading, UK

**Keywords:** evaluation, propensity score matching, doubly robust estimation, quasi-experimental design, higher education, transition

## Abstract

This study demonstrates how to evaluate a university-wide online course designed to support student transition into university by using Propensity Score Matching (PSM) and Doubly Robust Estimation (DRE). Using data from seven academic years, from 2016/17 to 2022/23, with more than 28,000 students, we examine whether enrolment in this optional pre-arrival course affects first-year pass rates. We also conducted additional analyses to compare outcomes from the year before and after the course’s implementation, as well as to examine these patterns across recent cohorts to potentially account for contextual changes over time. Results indicate that enrolled students show a 6.2 percentage point increase in the likelihood of passing Year 1, controlling for factors including sex, domicile, age, ethnicity, disability and socioeconomic status. We demonstrate how utilising existing institutional data can potentially strengthen evidence of impact for centralised initiatives and conclude with reflections on the use of such institutional data and matching techniques and their viability for future evaluations.

## Introduction

Transition to university is a critical phase that substantially affects students’ experience and success (Tinto, 1993). Universities deploy a range of interventions, from structured induction programmes to targeted transition events, to assist students in adapting to the new academic environment (Briggs et al., 2012). One approach has been to equip and prepare students prior to their arrival with academic study skills (Wingate, 2007), typically delivered online for greater engagement.

The aim of this paper is twofold. First, to determine whether enrolment in a pre-arrival online transition course would improve students’ pass rate at the end of the first academic year. Second, to demonstrate the potential of advanced statistical matching methods in the evaluation of higher education initiatives, such as Propensity Score Matching (PSM) and Doubly Robust Estimation (DRE), employed here as examples of a quasi-experimental design (QED) approach, by making use of existing institutional data to provide stronger comparative statistical evidence of impact. This study highlights the potential of using large institutional datasets that have previously been underutilised, and contributes to the relative lack of evaluation studies on university-wide educational interventions that use advanced statistical matching approaches (TASO, 2024). In England, there has been a renewed interest and emphasis on evaluation, especially causal evidence, by the regulatory body for higher education, as seen in the access and participation plans (APPs) that aim to set out how universities plan to improve access and success for underrepresented student groups (OfS, 2023).

This study aims to provide an applied example by documenting and reflecting on the processes of using these statistical methods. We demonstrate how existing student data already gathered by universities can be utilised to improve the evaluation of higher education through statistical matching techniques, and produce evidence of promise that is more in line with claims of causality (Blankenberger et al., 2021; Kaur et al., 2019; Powell et al., 2020; Reynolds & DesJardins, 2009). Through the methods of PSM and DRE, we reflect on the strengths and potential of institutional data, as well as the practical challenges and caveats.

## Supporting Student Transition Into University

Transition into university is a key phase where students experience and learn how to navigate their higher education, which subsequently shapes their engagement and integration into university life (Briggs et al., 2012). Yet the success of this transition can depend on students’ prior experiences and expectations. Previous research highlight that students who drop-out are mostly dissatisfied about their choice of study, often due to insufficient course information or due to a mismatch in expectations (Behr et al., 2020; Wong & Chiu, 2021). To reduce student attrition, it is important that students are supported and prepared for their journey into higher education (Tinto, 1993).

### Transition Activities and their Importance

As students enrol in higher education with different starting points and knowledge, their sense of self and approaches to learning will also inevitably vary (Leese, 2010; Wong, 2018; Wong & Chiu, 2019). Not all students will adhere to or resemble the ‘traditional’ backgrounds and traits of the typical university student, and the diversity of prior experiences and identities will mean there is a variety of ways in which students begin to navigate and negotiate their degree study (De Clercq et al., 2017). While universities support the transition of their students differently, these provisions tend to include a mixture of programmes such as a welcome or induction week or course, social events and the student union, university tours and academic support provisions, and mentoring schemes. The provisions aim to ease the transition process, reduce anxiety and promote confidence and a sense of belonging as students adapt to the university environment. A positive transition experience, even if there were challenges, can be key for students to continue at university (Brooman & Darwent, 2014).

### An Overview of the Theoretical Foundations of Student Transition

Transition into university is often more than just a change of place (Nora, 2001), but rather a personal journey of change into an environment that may be new, unfamiliar or even daunting, especially for those limited or no prior understanding of higher education. The writings of Bourdieu (1986), especially on cultural capital and habitus, provide a lens to understand these differences in student experiences. Bourdieu argued that those from privileged backgrounds are more likely to possess cultural capital, such as knowledge and know-how, that are valued at universities more than their less privileged counterparts. These individuals may have greater exposure to and knowledge about how university works, such as through family members, which in turn can generate greater confidence, sense of familiarity or even entitlement that others may lack. Bourdieu’s (1977) theory of habitus is also relevant: that is, the dispositions that shape how individuals perceive, respond to and react to their surroundings, including how well they feel they ‘fit in’ at university (e.g. Reay et al., 2010).

In other words, the habitus shapes what feels normal for individuals – at home, at school and at university. If there is an alignment of values between these domains, or fields, then students will likely find their values and available capital to be in sync. However, when there is a mismatch between the norms of the university and the values of the student, then there could be differences in how students typically approach their learning and how universities would expect their students to perform, behave or conduct themselves (Leathwood, 2006). A key purpose of university transition programmes is to pre-empt and make transparent to students the explicit and implicit rules and expectations within higher education, so that all students are able to benefit and maximise the support on offer and develop a sense of authentic belonging.

### Effectiveness of Transition Courses

There is evidence that students who complete a transition course have stronger continuation rates than those who do not, which is indicative of a positive correlation (Knox, 2005). Existing research suggests that transition courses that are longer are also more effective, such as those that last for an academic term, semester or even year, when compared to shorter courses, because students may experience different stages and phases of transition and adaptation in their first year of university (Christie et al., 2016).

The key components of successful transition courses seem to include those with a focus on academic study skills and literacy (Scouller et al., 2008), as ineffective study habits and predispositions can lead to disengagement and ultimately withdrawal (McKenzie & Schweitzer, 2001). According to Briggs et al. (2012), transition courses can be very effective when they are embedded into standard teaching, for example, covering study skills and assessment preparations. However, this approach may not work for everyone as Penn-Edwards and Donnison (2011) argued for the importance of a flexible model for transition support in recognition of how different students engage with support provisions.

Available literature suggests that student engagement with academic support is strongly linked to student continuation and attainment (Grillo & Leist, 2013; Tinto, 1993), although the methods used to evidence these claims have tended to lack data or analysis for claims of causality. As such, this paper contributes to a small but growing literature base on the use of advanced statistical matching techniques for higher education programmes designed to improve student outcomes and presents evidence of promise for causality. More specifically, we make use of a large multi-year institutional dataset to retrospectively evaluate the effectiveness of an optional online transition course on student continuation.

### Use of Matching Statistical Techniques in Education Research

A range of methods and methodologies are used in educational research to generate knowledge and evidence of impact. As a form of quantitative quasi-experimental approach, the use of statistical matching is often a practical method where a comparison is created between the treated and the untreated groups (Gorard, 2021). When randomisation is not viable or feasible for randomised controlled trails (RCTs), a matching approach focuses on available measurable or observable attributes, such as sex, socioeconomic status and ethnicity, as part of the control variables or baselines, to account for similarities between the different sample groups. The purpose is to reduce bias and improve the robustness of the findings (Reardon et al., 2019), although critical consideration is needed on the construction of comparable groups due the potential of selection bias when the samples are not random.

This paper utilises two advanced statistical methods: Propensity Score Matching (PSM) and Doubly Robust Estimation (DRE). PSM attempts to mimic randomisation by balancing observed characteristics between treated and untreated groups. DRE combines propensity scores with outcome regression models, offering greater protection against model misspecification and thus strengthening causal claims (Funk et al., 2011; Rosenbaum & Rubin, 1983; Stuart, 2010). These techniques are particularly valuable in higher education research, where true randomisation is often impractical or unethical. These methods allow more confident causal estimation from observational data. Although more established in fields such as medical research (Bai, 2011), PSM has been effectively applied in a growing number of higher education studies to assess causal relationships and impacts, particularly concerning access, retention and academic performance.

For instance, drawing on a sample of 2830 students at an Italian university, Agasisti et al. (2022) employed PSM and found that students who completed an online foundation course in physics were significantly more likely to succeed in subsequent exams, by 7–16 percentage points. This study is comparable with the present study as we aim to evaluate the effectiveness of a pre-arrival transition course on them passing the first year. Similarly, Bessudnov et al. (2015) used PSM to evaluate a European postdoctoral programme in the social sciences and reported positive effects on life satisfaction and research output amongst their participants (*n* = 155). Addressing equity issues, Bottia et al. (2020) applied PSM to a large sample (*n* = 19,640) of North Carolina community college students. Their results showed these students are likely to pursue and succeed in STEM fields regardless of socioeconomic status, providing evidence for the role community colleges play in improving STEM accessibility. Caviglia-Harris (2022) used PSM to evaluate the effect of ‘Living Learning Communities’ in the US, and found students improvements in student retention and academic performance from a sample of 4179 students. In Taiwan, Lau et al. (2014) found that participation in extracurricular activities for business school students (*n* = 28,768) to boost their employability skills, such as leadership and creativity. These studies suggest that while statistical matching methods can provide robust insights across various domains of higher education, their adoption remains limited and underutilised in much of the literature.

This study adds to the growing literature by applying PSM and DRE to evaluate an online transition course in a UK university setting. While earlier studies have shown the potential of these methods to explore academic and social outcomes (Agasisti et al., 2022; Bottia et al., 2020), their use in evaluating transition programmes within UK higher education remains limited. Drawing on a large institutional dataset, this paper offers a methodologically grounded assessment of impact and contributes to wider debates about causality in evaluation research. In doing so, we hope to provide a practical example for those in the sector who are seeking to make better use of existing institutional data, and to promote greater transparency in how such methods can be applied.

## The Study

### Context: The Transition Course

The transition course is a free online course created for new undergraduate students by the case-study university, with the aim to better prepare students for higher education study. It was launched in 2017/18, with gradual and incremental changes over the years in response to feedback. The course is available and advertised to all new students prior to the official start of their degree programmes and remains open for the first semester/term. It is optional but students are actively encouraged via email to take part. Academic tutors are also supported to remind and promote the transition course to their students. The course can typically be completed in 9 hours, with asynchronous videos and resources alongside an online forum with current students and course educators. It is designed so that students can work through it at their own pace, and it is hosted on a popular digital education platform. The course focuses on three main areas of university study: academic integrity, communicating at university, and independent learning. The aim is to help students understand how these concepts apply to their studies, including advice on engaging in seminars, getting the most out of their degree programme and using digital tools.

For context, the case-study university is a medium-sized English university with a student population and demographic composition that broadly reflects the national population (between 10,000 and 20,000 students). The case-study institution is neither extreme nor atypical in terms of student diversity and outcome, with a range of departments and disciplines.

### Research Question

*Research question*: Does enrolment in an optional online transition course impact students’ outcome, specifically passing or not passing Year 1?

Hypothesis Students who enrol in the transition course are more likely to pass Year 1 compared to those who do not enrol.

### Institutional Data

We used anonymised institutional data collected by the case-study university (through the Higher Education Statistics Agency, HESA). The dataset included first-year student records for the academic year before the transition course was implemented (2016/17), as well as the six years after (2017/28 to 2022/23), totalling 28,372 undergraduates over seven academic years, averaging 4000 students per cohort. The dataset include two key variables that marked (1) whether students enrolled in the optional university-wide transition course, and (2) whether the student passed Year 1 (at first or second attempt) at the end of their respective academic year. A range of demographic variables were provided, especially those marked as ‘widening participation’ data (see below). Due to restricted access to institutional data and data protection processes, the dataset we received and used only contained the information of interest, namely, whether the student enrolled in the transition course, whether they passed Year 1, and selected data about their demographic backgrounds (mostly provided as binary values). We are informed that the internal matching process used student ID and email. It is noted that the data we used are commonly collected by UK higher education providers, as per requirements or preparations for various data returns to regulatory bodies, which means the analysis we present should be highly replicable. Of course, individual institutions may vary on the breadth and completeness of the student data collected. The project received institutional ethics approval (Institute of Education, July 2024) and all data and codes used in this study are available in the researchers’ university data repository, accessible to users in accordance with repository guidelines (https://doi.org/10.17864/1947.001336). The provider of the data granted permission on the use and publication of the anonymised data in this retrospective non-interventional study.

As such, no individuals are identifiable and our presentation of data, including the breakdown of these variables (see Table 1), had sufficiently large numbers to ensure anonymity and reliable statistical analysis. Given our large sample size (*n* = 28,372), our study was well-powered to detect even small effect sizes. Our power analysis (using G*Power for logistic regression) indicated that we have over 99% power to detect a small effect size (Cohen’s d = 0.2; odds ratio = 1.20) at the 0.05 significance level. This level of power ensures our study can detect differences between variables, even modest differences, which are still meaningful in educational contexts (Cohen, 1988).

**Table 1.** Descriptive Overview of Dataset (*n* = 28,372).

Variable	Category	Frequency	%
Sex	Female	15,275	53.8
	Male	13,097	46.2
Domicile (UK)	No	4919	17.3
	Yes	23,453	82.7
Mature student	No	26,258	92.5
	Yes	2114	7.5
Minority ethnic student	No	18,292	64.5
	Yes	10,080	35.5
Disability declared	No	22,346	78.8
	Yes	6026	21.2
Disability student allowance	No	26,986	95.1
	Yes	1386	4.9
Household income < £25K	No	20,392	71.9
	Yes	7980	28.1
POLAR quintiles 1&2	No	23,212	81.8
	Yes	5160	18.2
Passing year 1	No	3450	12.2
	Yes	24,922	87.8
Enrolment in transition course	No	13,605	48.0
	Yes	14,767	52.0

As described later, we used the statistical techniques Propensity Score Matching (PSM) and Doubly Robust Estimation (DRE) to control for differences between students who did and did not enrol in this transition course, using the range of available variables.

### Variables

#### Treatment Variable

Enrolment in the transition course was our treatment variable, as measured by institutional records of whether the student had enrolled in the course. The standard practice is to code those treated (i.e. enrolled) as 1, and the control (i.e. not enrolled) as 0. As discussed later in the reflection, due to the treated group being in the majority, some of the processes were less standard, which is noted.

#### Outcome Variable

We are interested in whether students passed Year 1, as measured by institutional records of their continuation data, which we use to determine the extent to which this outcome (i.e. passing Year 1) is influenced by whether students had enrolled in the transition course.

#### Control Variables

The institutional dataset included a range of demographic variables, which are used as the control variables in the analysis to ascertain and isolate the impact of enrolment in the transition course on passing Year 1. The demographic data we used included sex (female, male), domicile (non-UK, UK), age (non-mature, mature), ethnicity (non-minority ethnic, minority ethnic students), disability declaration (no, yes), disabled students’ allowance (no, yes), household income (above £25,000, below £25,000), participation of local areas (POLAR) classification (Quintiles 3, 4 & 5; Quintiles 1 & 2, where the lower quintiles represent those areas with the lowest proportion of young people entering higher education). These eight variables were available as binary values, with smaller or incomplete entries omitted. With the exceptions of *sex* and *domicile*, the other six variables are part of the ‘widening participation’ markers commonly and historically used as indicators of inequalities in access and participation work, at least within the acquired dataset.

These variables are theoretically grounded in existing literature as key factors that shape students’ educational experiences and outcomes. Our selection aligns with prior research indicating these variables influence continuation and success in higher education (Grillo & Leist, 2013; Leese, 2010; Tinto, 1993). For instance, in sector-wide equality reports (e.g. Advance HE, 2024), student demographics such as gender, socioeconomic status, ethnicity, disability and age are often reported and linked to outcomes. Empirical studies have consistently documented how these identities intersect with structural inequalities to produce varied educational outcomes (Burke, 2012; Mountford-Zimdars et al., 2015). It is also recognised that some of these markers are more tailored towards UK home students, in terms of data availability and accuracy, such as household income, disability student allowance and POLAR (see also Jones et al., 2017). We included *sex* and *domicile* in our analysis as the data were available and we recognise their potential influences on student enrolment in an optional transition course as well as their end of year pass rate.

## Provisional Analysis

This section outlines the analytical process used to assess how the transition course affected passing Year 1. After comparing the pass rates of enrolled and non-enrolled students using a descriptive analysis, we use a *t* test to determine whether the differences are statistically significant. Finally, multivariable logistic regressions are used to assess the odds of passing Year 1 as well as enrolment, controlling for key confounding variables, which provide a more robust understanding of how different factors shape passing Year 1 and enrolment in the transition course.

### Descriptive Analysis of Passing Year 1

Table 2 provides a straightforward comparison between passing Year 1 (outcome) and enrolment in the transition course (treatment). Since the course’s introduction in 2017/18, there has been a persistent pass rate gap between enrolled and non-enrolled students, averaging 7 percentage points over six academic years (2017/18 to 2022/23). The overall baseline pass rate across this period was 87.4 %, with non-enrolled students passing at 83.2 % compared to 90.2 % for enrolled students.

**Table 2.** Transition Course Enrolment and First-Year Pass Rates by Academic Year.

Academic year	Year 1 Cohort	% Enrolled in Course	Non-Enrolled Pass (%)	Enrolled Pass	Pass Gap	Overall Pass (%)
2016/17	3962	0	90.5	N/A	N/A	90.5
2017/18	4332	56.6	83.8	90.3%	6.5 pp	87.5
2018/19	3943	67.5	78.0	88.8%	10.8 pp	85.3
2019/20	3852	59.2	90.2	93.2%	3.0 pp	92.0
2020/21	3920	59.5	85.5	92.2%	6.7 pp	89.5
2021/22	4069	58.6	79.7	88.5%	8.8 pp	84.9
2022/23	4303	61.9	81.2	88.5%	7.3 pp	85.7

*Note.* pp = percentage point.

### *t* test

A two-sample *t* test, excluding data from 2016/17 (*n* = 24,411, 2017/18 to 2022/23), confirmed the above difference as statistically significant (95% CI: 0.061, 0.078; t (24,409) = −16.112, *p* < .001), suggesting that enrolment in the transition course is associated with a higher pass rate. The *t* test compares the average pass rates of students enrolled and not enrolled in the transition course, which highlighted statistically significant differences over 6 years.

It is noted that the pass rate in 2016/17, the year before the transition course was introduced, is amongst the highest (90.5 %, second to 2019/20, with 92.0 %). This will be revisited in the discussion; for now, it is clear that, since the course introduction, enrolled students consistently demonstrate higher pass rates.

### Multivariable Logistic Regression

To further understand the factors that influence students’ likelihood of passing Year 1 and enrolling in the transition course, we carried out separate multivariable logistic regressions on data from 2016/17 (*n* = 3692) for passing Year 1, as well as data from 2017/18 to 2022/23 (*n* = 24,411) for both outcomes. Using the combined data from 2017/18 to 2022/23 will enable a more robust analysis of the impact of the transition course across multiple cohorts. Multivariable logistic regression is used to identify predictors of passing Year 1 or enrolling in the transition course, while controlling for multiple covariates. This approach estimates the odds of success for each factor and accounts for their interactions and combined effects on the outcome. For these regressions, a Hosmer-Lemeshow goodness-of-fit test also indicated a good fit between the observed and expected outcomes.

Table 3 shows the pass rate for the 2016/17 cohort, prior to the introduction of the transition course, while Table 4 focuses on data from 2017/18 to 2022/23, which include enrolment in the transition course within the predictive model for passing Year 1. Although this additional variable means both tables are not directly comparable, the baseline data in Table 3 provides a useful reference point that illustrates how these different variables predict their likelihood of passing Year 1. For example, male (OR = 0.720), ethnic minority (OR = 0.670) and mature (OR = 0.507) students had significantly lower odds of passing Year 1 than their respective reference groups, namely, female, non-minority ethnic and non-mature students. Similar patterns can also be seen in Table 4, although it is clear that students who enrolled in the transition course had significantly higher odds of passing Year 1 (OR = 1.772) than those who did not, as did students who received a Disability Student Allowance (OR = 1.474), as statistically significant predictors since the introduction of the transition module.

**Table 3.** Multivariable Logistic Regression Analysis Predicting Passing Year 1 (2016/17).

Variable	B (Estimate)	Exp (B)	95% CI Lower	95% CI Upper	*p*-Value
Sex (male)	−0.327	0.720	0.581	0.892	0.003
UK domicile	0.107	1.113	0.832	1.485	0.471
Mature student	−0.679	0.507	0.360	0.717	<.001
Ethnic minority	−0.402	0.670	0.529	0.845	0.001
Disability declared	0.019	1.018	0.763	1.360	0.899
Disability student allowance	−0.055	0.947	0.362	2.473	0.911
Household income < £25K	−0.257	0.774	0.590	1.013	0.061
POLAR quintiles 1&2	−0.076	0.926	0.701	1.224	0.592
Constant	2.485	12.998	9.630	17.543	<.001

*Note*^1^: B (Estimate) = Unstandardised regression coefficient, Exp(B) = Odds Ratio, CI = Confidence Interval.

**Table 4.** Multivariable Logistic Regression Analysis Predicting Passing Year 1 (2017/18 to 2022/23).

Variable	B (Estimate)	Exp (B)	95% CI Lower	95% CI Upper	*p*-Value
Sex (male)	−0.266	0.767	0.710	0.830	<.001
UK domicile	−0.312	0.733	0.652	0.823	<.001
Mature student	−0.411	0.664	0.584	0.755	<.001
Ethnic minority	−0.356	0.701	0.647	0.760	<.001
Disability declared	−0.064	0.938	0.845	1.041	.226
Disability student allowance	0.388	1.474	1.195	1.819	<.001
Household income < £25K	−0.294	0.746	0.686	0.811	<.001
POLAR quintiles 1&2	−0.186	0.830	0.754	0.913	<.001
Enrolment in transition course	0.572	1.772	1.639	1.915	<.001
Constant	2.320	10.203	8.958	11.620	<.001

*Note.* B (Estimate) = Unstandardised regression coefficient, Exp(B) = Odds Ratio, CI = Confidence Interval.

Table 5 provides an analysis of the factors predicting enrolment in the transition course, which are relatively similar to the patterns observed in Table 4 in terms of passing Year 1. Again, the groups with significantly lower odds of enrolment are also male (OR = 0.498), ethnic minority (0.846) and mature (0.789) students. An interesting difference is UK-domiciled students, who had higher odds of enrolment in the transition course (OR = 1.522) but lower odds of passing Year 1 (OR = 0.733, see Table 4) than non-UK-domiciled students.

**Table 5.** Multivariable Logistic Regression Analysis Predicting Enrolment in Transition Course (2017/18 to 2022/23).

Variable	B (Estimate)	Exp (B)	95% CI Lower	95% CI Upper	*p*-value
Sex (male)	−0.696	0.498	0.473	0.526	<.001
UK domicile	0.419	1.522	1.413	1.638	<.001
Mature student	−0.237	0.789	0.715	0.871	<.001
Ethnic minority	−0.168	0.846	0.799	0.894	<.001
Disability declared	−0.023	0.977	0.909	1.050	.529
Disability student allowance	0.248	1.282	1.120	1.467	<.001
Household income < £25K	−0.059	0.943	0.888	1.002	.057
POLAR quintiles 1&2	0.021	1.021	0.953	1.094	.552
Constant	0.503	1.655	1.533	1.787	<.001

*Note.* B (Estimate) = Unstandardised regression coefficient, Exp(B) = Odds Ratio, CI = Confidence Interval.

While these patterns merit further investigation, including the interaction of variables, it is important to note that the pseudo-R squares (a proxy measure of model fit) for these models were 0.020 (Table 3) and 0.029 (Tables 4 and 5), indicating that the models explain only about 2.0%–2.9% of the variance. A low R squares value suggests that there are other (unmeasured) variables influencing student progression, which are not captured by the current models; we are limited by the data we can capture about the students. The fact that some of the same variables predict both enrolment in the transition course and passing Year 1 suggests that any apparent effects of enrolment the transition course might instead be due to confounding factors such as sex or other demographic variables. These limitations highlight the value for more robust statistical techniques, such as Propensity Score Matching (PSM) and Doubly Robust Estimation (DRE), which can better account for shared predictors and adjust for confounding variables to strengthen causal interpretation.

## Statistical Matching Methods

We used Propensity Score Matching (PSM) and Doubly Robust Estimation (DRE) to control for outcome differences (i.e. passing Year 1) between students who did and did not enrol in the transition course. PSM is a statistical technique used to reduce selection bias by equating groups based on selected covariates (or control variables), which allows the research to estimate statistically the treatment effect of an intervention while accounting for confounding variables (Rosenbaum & Rubin, 1983; Stuart, 2010). In this study, PSM creates comparable groups by matching students who enrolled in the transition course (treated) with those who did not (control), based on their propensity of enrolment. Recognising the limits of PSM, including the assumption that all relevant variables have been measured and correctly specified (Pearl, 2009), we also incorporated DRE to safeguard against model misspecification (Uysal, 2015), which can occur when a statistical model fails to accurately represent the relationships within the data or omits relevant variables, which can potentially lead to biased or incorrect conclusions (Funk et al., 2011).

This dual approach contributes to a methodological innovation in higher education evaluation and provides more robust and reliable findings. While the use of DRE is not as common in educational research (Liu & Borden, 2019), it is valuable to apply alongside PSM to strengthen the robustness and reliability of research outcomes. As discussed below, to ensure robust matching, we assessed the covariate balance before and after matching and used a low calliper to strengthen the matching process and minimise bias.

### Matching Technique

Matching is an approach used to pair treatment and comparison groups that have similar measurable traits (Bai, 2011). As such, we initially used data from 2017/18 to 2022/23 (*n* = 24,411). We applied a calliper matching on the propensity score, with a conservative range of 0.01, which tends to yield better performances than higher ranges for estimating treatment effects (Austin, 2009). This approach limits the matching to a maximum difference of 0.01 on the propensity score between students who have and have not enrolled in the transition course, ensuring a lower match tolerance of variability. The propensity score was calculated using the eight covariates (see Table 6). Additionally, the results were similar when a larger width of callipers (0.02, 0.05, 0.10) were tested, indicating that our analyses are robust to variations in matching strictness (Wang, 2021). Wider callipers (e.g. 0.05) tend to produce larger matched samples but poorer covariate balance, with a higher standardised mean differences across the variables. Given the large dataset, the chosen calliper width of 0.01 provided the best covariate balance across all variables while retaining a sufficient matched sample size for analysis (see Table 6). Our matched sample closely reflected the treated and control groups’ characteristics, which reduces the likelihood of bias due to poor matching quality. We also observed a sufficient overlap (or strong ‘common support’) between the controlled and treated groups, which meant the matching is consistent across the breadth of propensity scores (ranged from 0.343 to 0.763) and that there are comparable control and treated units for meaningful comparisons. In Stata’s *teffects* command, the default nearest neighbour matching pairs each treated observation with the control observation that has the closest propensity score, ensuring the most similar comparison based on observed covariates.

**Table 6.** Covariate Balance Summary Before and After Matching (Treated vs. Untreated).

Variable	Standardised Differences (Raw)	Standardised Differences (Matched)	Variance Ratio (Raw)	Variance Ratio (Matched)
Sex	−0.349	<0.001	0.978	0.999
UK domicile	0.180	<-0.001	0.737	1.002
Mature student	−0.048	<-0.001	0.859	0.997
Ethnic minority	−0.115	<0.001	0.942	0.999
Disability declared	0.065	0.001	1.096	1.001
Disability student allowance	0.085	0.001	1.414	1.005
Household income < £25K	0.017	<0.001	1.015	1.000
POLAR quintiles 1 & 2	0.041	<0.001	1.070	1.000

*Note.* Standardised differences and variance ratios are presented for both raw and matched samples.

### Assessing Balance

**Figure 1.**
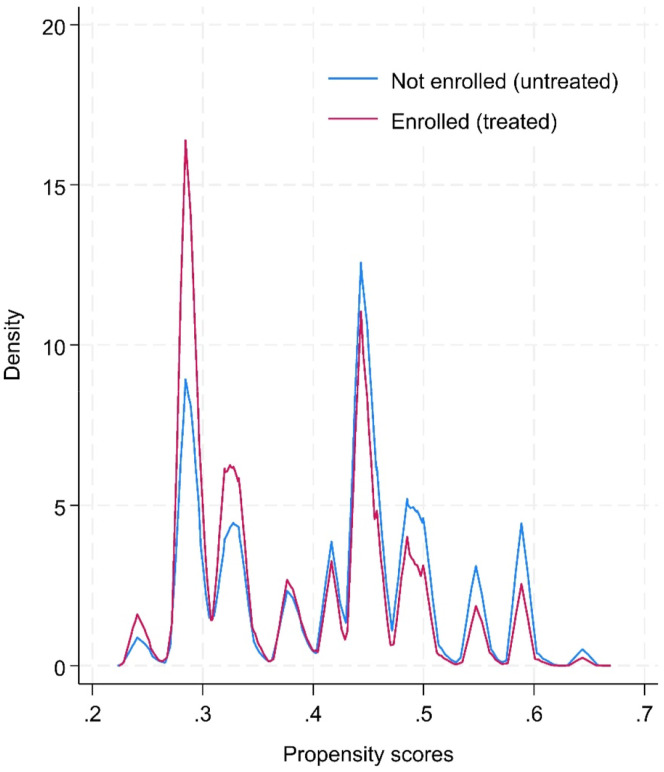
Overlap of propensity scores between treated and control groups.

Covariate balance was assessed before and after matching. Before matching, statistically significant differences were observed between the treated and untreated sample. This strengthened the rationale for a PSM to create more comparable groups. As indicated in Table 6, differences in propensity scores by sex showed a significant difference before matching (standardised mean difference, SMD = −0.349). After matching, the SMD for sex was reduced to <0.001. SMD is a measure of the effect size between two groups, after accounting for potential confounding variables, similar to Cohen’s d. A smaller SMD indicates a smaller difference between groups relative to within-group variability, suggesting a more balanced distribution of covariates between the treatment and control groups. After matching, the covariates were well-balanced, with standardised biases reduced to near zero, confirming that the PSM procedure successfully created comparable groups. The overlap plot (see Figure 1) also demonstrates sufficient overlap in propensity scores between the treated and untreated groups, which further reinforces the validity of the matching process and supports the reliability of the estimated treatment effects. As such, the matched sample provides a robust basis for estimating the treatment effect by minimising the differences between the treated and control groups.

### Missing Data

In *Stata*, the *teffects* function automatically excludes any observations with missing values in the variables used in the propensity score model from the analysis. This process – listwise deletion – means that only units (observations) with complete data on all variables are included in the model for matching. It should be noted that students with values outside the binary recoding of 0 and 1 were already removed due to low numbers (e.g. the dataset only included Yes/No for enrolment in the transition course and seven covariates, with female/male for sex). Fewer than five records were removed in the analyses below.

### Propensity Score Matching (PSM) Results

We used *Stata* software to carry out the PSM and tested both the built-in *teffects* function and the *psmatch2* package (Leuven & Sianesi, 2003). The *teffects* function estimates the treatment effects by creating matched samples and controlling for confounding variables, allowing for causal inference from observational data. The *psmatch2* package provides options for matching algorithms and diagnostics, adding flexibility and depth of the PSM analysis. While we used *teffects*, being the native function, elements of the *psmatch2* function were also applied, such as logistic regression and the saving of propensity scores. Through these tools, we reduced our eight confounding control variables into a single propensity score ranging from 0 to 1 using logit regression. Here, the propensity score represents the conditional probability of a student enrolling in the transition course. The treatment effect, or the impact of enrolment in the transition course on passing Year 1, was estimated using the matched samples.

The results revealed a significant positive effect of enrolment in the transition course on passing Year 1, indicating that students who enrolled were more likely to pass their first year compared to those who did not, controlling for the eight variables in the model. As with the provisional analysis, data from 2017/18 to 2022/23 were collated, which also help to balance out annual fluctuations (e.g. 2019/20, during the COVID pandemic). As explained below, we conducted additional analyses that also made use of specific cohorts to attest different matching approaches.

Students who enrolled were 6.3 % more likely to pass Year 1 according to the Average Treatment Effect (ATE: coefficient = 0.063, SE = 0.005, z = 13.80, *p* < .001, 95 % CI [0.054, 0.072]), while the Average Treatment Effect on the Treated (ATET) indicates a 6.2 percentage points increase (coefficient 0.062, SE = 0.005, z = 13.19, *p* < .001, 95% CI [0.053, 0.071]). The average treatment effect (ATE) measures the expected difference in outcomes if the entire population were to receive the treatment (i.e. enrolled) compared to if the entire population were not to receive the treatment, while the Average Treatment Effect on the Treated (ATET) measures the expected difference in outcomes for those who actually received the treatment compared to what their outcomes would have been had they not received the treatment (StataCorp, 2023). In other words, ATET focuses specifically on the effect of the treatment for those who were treated. Both results are very similar, showing that enrolment in the transition course is positively related to passing Year 1.

To strengthen the robustness of these findings, we conducted two additional analyses. The first included the 2016/17 dataset as a new control group, the year before the launch of the transition course. This approach in theory provides a truer control as students did not have the option to partake in the transition course, although we acknowledge a time difference which meant there could be variations in student provisions or access to support systems when compared to the following year when the course was launched. Yet this approach offers an alternative and arguably clearer baseline for comparison by further reducing unaccounted confounders such as student motivation and likely academic outcomes (regardless of enrolment in the transition course) that could impact their performance. The first additional analysis included 6410 observations after matching, using the 2016/17 cohort (*n* = 3961) but only those who enrolled in the transition course in the 2017/18 cohort (*n* = 2449). Repeating the PSM processes as described above, this additional analysis resulted in an unexpected outcome, where enrolment in the transition course was associated with a very small and statistically non-significant negative effect on passing Year 1 (ATE = −0.003, SE = 0.008, z = −0.47, *p* = .641, ATET = −0.003, SE = 0.008, z = −0.43, *p* = .666). It is noted that the pass rate for the 2016/17 cohort was already higher at 90.5%, compared to an average of 87.4% from 2017/18 to 2022/23 for all students, with a rate of 90.2% for the enrolled and 83.2% for those not enrolled. The pass rate for students enrolled in the transition course in 2017/18 was 90.3%. Changes in pass rate may or may not also reflect the shift in student profiles or populations at the case-study university, especially with the persistent pass rate gap of 6.2 percentage points (for ATET) between those who did and did not enrol in the transition course since its inception.

The second additional analysis attempted to control for student prior attainment through the variable UCAS entry tariff points, using data from specific cohorts as a test. This variable had numeric values that corresponded to the points equivalent of school-level qualifications, such as A-level, and is thus a marker of previous academic achievement. It was, however, absent in our main analysis above because the data in this variable were inconsistent and incomplete across cohorts (see also Discussion section). In this pilot, we focused on the most frequent values for UCAS entry tariff points as a limited test to include prior attainment as a control variable. We constructed a viable sample using the 2021/22 and 2022/23 cohorts, where the most frequent UCAS entry tariff points – 92, 112, 120, 128, 136, 144 – all with over 200 students for each value, constituted just under half of the entire cohorts in 2021/22 (*n* = 1,850, 45.2%) and 2022/23 (*n* = 2,015, 46.8%). For information, 57.1% and 63.3% of students in the subset enrolled in the transition module, comparable to their respective main cohort (see Table 2). The test revealed that the gap in Year 1 pass rates between those enrolled and not enrolled in the transition course increased to between 7.01 and 7.20 percentage points. Similar tests were piloted using earlier cohorts, especially 2016/17 and 2017/18, but the results were statistically non-significant. In short, our limited test indicates that even when controlling for student prior attainment, the impact of enrolling in the transition course on passing Year 1 still holds.

Despite these results, it remains crucial to consider these findings within the broader context of continuous changes in student demographics, university policies and wider social or global issues.

### Doubly Robust Estimation (DRE) Results

We validated our PSM results using the doubly robust estimation (DRE) approach, which combines outcome regression and propensity score weighting to strengthen causal inference. We used the augmented inverse probability weighting (AIPW) command in *Stata* (*teffects aipw*) to estimate the treatment effects. To generate doubly robust estimates, the AIPW method combines outcome regression and propensity score weighting so that only one model needs to be correctly specified for the estimates to remain unbiased (Bang & Robins, 2005). The outcome model was specified as a linear regression with the same covariates included as in the propensity score model. From the earlier analysis (*n* = 24,411), the average treatment effect (*ATE*) of participating in the transition course was found to be highly significant.

Consistent with those results, the analysis showed that enrolment increased the probability of passing Year 1 by approximately 6.3 percentage points (ATE = 0.063, SE = 0.005, z = 13.98, *p* < .001, 95 % CI [0.054, 0.072]), suggesting a robust positive effect of the transition course on student outcomes. In addition to the ATE, the mean outcome for the control group (students who did not participate in the transition course) was estimated, with the propensity score mean (*POmean*) for the control group being 0.836, indicating that on average, 83.6% of students in the control group passed Year 1. This estimate was significant (SE = 0.004, z = 219.20, *p* < .001), with a 95% confidence interval ranging from 82.9% to 84.4%.

DRE for the additional analyses was also conducted. For the first post-test, using the whole 2016/17 but only 2017/18 data of those enrolled (*n* = 6410), the result for enrolment and passing Year 1 was statistically non-significant (ATE = −0.003, SE = 0.008, z = −0.48, *p* = .629). The propensity score mean for the control group was 0.905 (SE = 0.004, z = 196.08, *p* < .001, 95 % CI [0.897, 0.915]), which was statistically significant and indicate that on average, 90.5% of students in the control group passed Year 1. This high baseline for the control group, before the course was introduced, may explain why the course effect was not observable.

For the second additional analysis, using selected data from the 2021/22 and 2022/23 cohorts, the average treatment effect (ATE) indicated a 7.2 percentage point increase in the probability of students who are enrolled in the transition course to pass Year 1, and this effect was statistically significant (ATE = 0.072, SE = 0.012, z = 5.93, *p* < .001, 95 % CI [0.084, 0.096]). The propensity score mean for the control group estimated that on average 80.7 % of students not enrolled in the transition course passed Year 1, which was also statistically significant (SE = 0.010, z = 79.90, *p* < .001, 95% CI [0.787, 0.826]). This lower baseline pass rate for non-enrolled students suggests that the transition course may play a more pronounced role in supporting students to pass Year 1 in recent years.

In summary, the results from the DRE provided nearly identical results, which provides reassurance about the robustness of the PSM approach employed.

## Discussion and Conclusion

The use of Propensity Score Matching (PSM) and Doubly Robust Estimation (DRE) enabled us to assert robustly that for students in our case-study university, enrolment in this university-wide optional online transition course makes a positive difference to their likelihood of passing Year 1 (Grillo & Leist, 2013). We initially found this difference to be 6.2 percentage points, after controlling for and matching on student demographic backgrounds, such as sex, ethnicity, age, disability and socioeconomic status. Given this paper focuses on the use of statistical matching methods in higher education evaluation, our discussion below will reflect on the challenges, opportunities and potential implications of this approach.

### Contextualising the Results

If we apply this 6.2 percentage points improvement gap to all students who did not enrol in the course (*n* = 9643) when they could have, we estimate that approximately 598 additional students would have passed Year 1 if they had enrolled (around 100 students per academic year). This counterfactual can assume that, had all non-enrolled students taken the course, they would have experienced the same benefit. The transition course thus has the potential to significantly boost overall pass rates amongst non-enrolled students. From a policy perspective, this gain has practical importance as it improves student retention and therefore institutional metrics on student continuation as well income from tuition fees. More broadly, the findings remind institutions to invest strategically in support of transition initiatives for students, especially for those who may experience greater inequalities of opportunity.

We recognise that our result of a 6.2 percentage points gap between the pass rates of those who enrolled and not enrolled in the transition course can be difficult to contextualise in the absence of a widely accepted baseline. As such, perhaps our finding will provide a starting point for others to compare and contrast (Cook & Steiner, 2010). We acknowledge that this counterfactual should be viewed as an initial benchmark for further investigation. Furthermore, our observed gap closely aligns with the 7 to 16 percentage point difference reported by Agasisti et al. (2022) in their PSM study of a foundation physics course in Italy. Our research, however, does not answer the question of whether the gap we identified is ‘big enough,’ which may require a detailed cost-benefit economic evaluation (Dynarski, 2003). That said, institutions who wish to improve their student continuation and success may find evidence from our findings that pre-arrival transition courses can work, and to ensure that it benefits all students, these should be fully embedded across official academic and support provisions.

However, we want to preface that our outcome variable was passing Year 1, and what happens in Year 2 and beyond is not currently captured. The longer-term impact of the transition course will be more difficult to control or measure due to the growing range of variables and depth of influences over time, which inevitably reduces the strength to claims of impact. As such, our focus limits to an academic year (i.e. from enrolment at the beginning to passing Year 1 at the end), but even with this shorter timeframe, there are still many factors our dataset does not capture.

Indeed, while institutional data is extensive, there are inherent limitations in that it tends to only include more static variables such as student demographic backgrounds, but less likely to consider factors such as student motivation, attitudes and experiences, all of which can significantly influence educational outcomes. These unmeasured factors will likely play a role them passing Year 1, or enrolling in the transition course, and are therefore important caveats and aspirational for future studies to capture and incorporate into the data and analysis.

Empirically, what the results do show is the value of targeted early interventions in enhancing student outcomes at university, which can be particularly important for students from underrepresented backgrounds (Wong, 2018). A strong foundation in academic study skills would better prepare students to maximise the opportunities and resources at their disposal (Wong & Chiu, 2019).

### Challenges in the Propensity Score Matching Process

PSM aims to provide robust causal evidence, but it is recognised that there are inherent limitations and caveats to this method (Shipman et al., 2017). While PSM and DRE strengthen causal claims by addressing confounding variables, these methods rely on the assumption that all relevant confounders are measured. This limitation means it is critical to have the robust covariates within the model, but such comprehensive set of data are not always available or possible, especially at scale, which means there are key factors of influences that could be missing from the analysis. As such, the analyses only provide a direct comparison for the available variables and cannot robustly account for confounders and motivations that are not recorded. Although our institutional dataset collected most demographic variables, there are also other factors of influence at the central and local levels that are not captured (Rosenbaum & Rubin, 1983), such as student self-efficacy, motivation, financial support or engagement in various activities or extracurriculars, all of which can play a direct or indirect role in student continuation or outcomes (e.g. Grillo & Leist, 2013; Lau et al., 2014). As revisited in the reflection below, prior student attainment in the form of UCAS entry point tariff is a data variable collected by the university, but there are inconsistencies in the data collected and it was therefore omitted from the analysis. It is also acknowledged that the data collected on enrolment in the transition course is also not the same as students’ engagement in the course, such that more granular data on course completion or early withdrawal, for instance, were unavailable. It is important to note that our analysis is based solely on a binary indicator of enrolment, which does not capture the degree of students’ engagement or course completion. While anecdotal evidence suggests high completion rates, the absence of detailed participation metrics represents a limitation. Future studies should incorporate such metrics to distinguish the impact of mere enrolment from that of active course participation. Although anecdotal evidence suggests a strong positive association between enrolment and completion in the transition course, the available data are crude in that most were in binary values and missing potential confounders is unavoidable. That said, the large sample size may reduce the random effect of unobserved variables, but this hypothesis requires further evidence (Austin, 2009; Morgan & Winship, 2014).

Another reflection relates to the matching process within PSM, which will most likely be of concern to activities (or treatments) that are centralised with a large uptake. Unlike the pattern where the control sample is typically larger than the treated sample, our treated group (i.e. those who enrolled) is larger than our control (those who did not enrol). We found that the default flow for PSM, in *Stata* and the *teffects* command at least, meant that the system aspired to provide a match for everyone in the treated sample (*n* = 14,768, coded as 1). Yet, with only 9643 students in the control sample (coded as 0), this imbalance resulted in students in the control group – within the boundaries of the propensity score – being matched more than once to the treated. In other words, the same control student was matched with more than one students in the treated sample, despite the default nearest neighbour setting of just one match (*nneighbor(1)*) within the *teffects* function. To crosscheck and mitigate this, we reran the PSM but reversed the treatment variable whereby those who did enrol was recoded as 0, while those who did not enrol was coded as 1 (Stuart, 2010). This resulted in the total observation of matches to be 19,286 students (where 9643 was the new treated and 9643 was the new control). After assessing the balance that had minimal differences in standardised mean differences, and therefore a robust match, the controlled difference was −6.2 percentage points, which once converted, yields very similar results. In short, this process helped to strengthen our original analysis, which ended up applying an inflated matched sample that could have challenged the robustness of the analyses (King & Nielsen, 2019). Future studies could also consider coarsened exact matching (CEM) as an alternative approach, which groups covariates into broader categories to reduce reliance on repeated matches while improving covariate balance.

This issue was not applicable in the additional analysis as the control group (entire 2016/17 cohort) was larger than the treatment group (selected 2017/18 cohort, only those enrolled). The additional analysis helped to add nuance to the interpretations and caveats to the data, especially as the results were statistically non-significant and the raw pass rate was actually higher than all subsequent years. A number of possible factors may explain for this discrepancy, including the characteristics of the cohort, changes in teaching and learning policies and provisions, such as the curriculum and academic support, as well as institutional resources allocations that coincided with the introduction of the transition course. Wider societal factors can also shape the holistic environment of the student experience during that period, although these are only speculations. These external factors highlight the need to situate findings within their broader institutional, societal and temporal contexts. In short, there are multiple factors that can shape student outcomes, beyond enrolment in the transition course alone. Consequently, the non-significant results highlight the inherent complexity of attempting to isolate the impact of the course when there are dynamic contextual factors of influence.

Regardless, we argue that the initial approach we showcased remains valid as using the treated and control groups from the same time period also helps to control for potential differences from one year to the next in terms of specific or temporary provisions or interventions. The persistent gap in passing Year 1 since 2017/18, suggests the presence of the transition course is now ever more important to at least maintaining the pass rate as seen back in 2016/17, before its introduction. It is also important to note that more historical pass rates may not be as meaningful for recent cohorts, given temporal changes over the years, especially with the pandemic and the cost of living crisis.

We are also wary that there are alternative or additional methods to PSM and DRE that function with a similar logic in terms of matched comparisons (e.g. entropy balancing). Therefore, the examples we presented remain just one of many ways to strengthen our claims regarding evidence of impact.

### Selection and Exclusion of Covariates

As inferred, the matching methods require a selection of covariates (or control variables) as markers to help build the profiles of student groups. Based on the type and completeness of our data, we included eight covariates. A ninth covariate, which we initially tried but was ultimately dropped for the main analyses was the *UCAS entry tariff point*, which can be seen as a proxy for students’ entry academic capability, as measured by their prior qualifications. In other words, the aspiration was to be able to control for prior attainment, so to speak, alongside the eight demographic variables. One of the working assumptions is that students who may benefit the most from such a transition course would be those whose prior attainment is lower, and if so, a specific course to strengthen academic study skills should, in theory, make a positive contribution to their end of year outcomes.

However, data on this variable was incomplete with 4076 missing values (from 24,411 students), most of whom were non-UK domicile international students, who presumably had qualifications that were not convertible into an equivalent *UCAS entry tariff point*. Furthermore, the values in this variable range from six to 504, considerably broader than the typical range expected of three A-level grades of, say, CCC (96 points), BBB (120 points) or A*A*A* (168 points). The variations recorded may reflect the changes in the UCAS entry tariff points system in 2017 which effectively lowered and changed the points conversion for all qualifications. In short, the data was inconsistent and the analysis proved difficult for meaningful interpretation, especially if this variable was also recoded into a binary (e.g. upper and lower 50%) for ease of generating comparable propensity scores alongside the existing covariates (Guo & Fraser, 2014). In the end, we decided not to include this variable for the main analysis and relied on the large samples to achieve a balance in the spread of prior attainment, in a similar spirit of a randomised controlled trial. However, we acknowledge that this approach may not completely randomise the allocation of the treatment group, due to the different reasons and motivations for student enrolling in the transition course or not. As already acknowledged, there will be unmeasured factors, such as student attitudes or experiences that can shape their engagement with the course, as well as the degree more broadly. To address this limitation, future research could employ sensitivity analyses, such as Rosenbaum bounds, to estimate the potential impact of unmeasured confounding on the treatment effect. Rosenbaum bounds provide an indication of how strong unmeasured confounding would need to be to invalidate the observed results, which would strengthen the robustness of causal claims (Rosenbaum, 2002). As the available data on prior academic performance is inconsistent, its use could bias our causal estimates, thereby highlighting the need for further sensitivity analyses in future research.

To reduce potential biases, we conducted additional analyses as crosschecks and post-tests. While not part of the original study design, they provide valuable supplementary insights that complement the primary analyses. Specifically, we reran the matching models using only the most common UCAS entry tariff points within the dataset, stratified by different academic year groups. These analyses offer additional insights into the role of prior academic attainment in students’ Year 1 outcomes following transition course enrolment. By focusing on cohorts with the most common UCAS entry tariff points, we aimed to create a more comparable baseline for examining the transition course’s impact across students with similar prior attainment. Significant differences observed for the recent cohorts (2021/22 and 2022/23), contrasted with the non-significant findings for the earliest cohorts (2016/17 and 2017/18), which reinforces the pattern that shows enrolment in the transition course is now more relevant than in the past in relation to students passing Year 1. Subject to more complete and consistent data, the variable on prior academic attainment warrants further investigation and inclusion in future evaluation and analysis of the transition course.

### Implications for Future Research and Evaluation

Given the added benefits we observed of the transition course in this study, future research could expand to assess the longitudinal impacts of such intervention. However, it is acknowledged that students’ spheres of influence will inevitably widen with time, which means the range of observable and unobservable factors will likely increase and become more difficult to control and measure (Smith & Todd, 2005). Yet considerable time and resources are invested to support student transition into university and the analysis of institutional data as presented here can help to bridge the gap in evidence that draws on large data, especially information that is already collected for regulatory purposes. Of course, the specifics will vary, as will the usability due to the quality, accuracy and completeness of data (Johnson & Wislar, 2012).

Future research and evaluation should include medium-term data such as pre- and post-activity questionnaires as a way to measure intermediate outcomes regarding the short and medium-term outcomes, such as student attitudes and experiences, which are assumed to lead to increased likelihood of passing Year 1 and beyond. Likewise, the analysis could look into cohort-specific trends for any potential temporal effects or anomalies in the transition course’s effectiveness over time. A deeper examination of cohort-level data may provide more nuanced insights into the changing needs of students and the institutional contexts, with opportunities for more targeted intervention design and evaluation. For context, such data collection is already underway for the forthcoming cohort of the transition course in question and is expected to provide more granular insights into the effectiveness of the intervention.

The scalability of this intervention to other institutions will depend on factors such as the availability of good-quality data on student demographics to inform both design and evaluation. While an online format increases accessibility, implementation tend to require significant upfront investment in course design and delivery platforms, as well as ongoing maintenance, staffing and evaluation expertise to ensure robust evidence of impact. Institutions with limited resources may benefit from collaborative partnerships or external funding to tailor such interventions to the needs of diverse students.

Further analysis could also probe into these differences by department or discipline. For instance, data from the case-study university show that over 5 years, enrolment rates in the optional transition course ranged from just under half of all students in some departments to almost three-quarters of students in other departments. There will likely be practices or lessons that could be shared to increase enrolment, for example. However, it is acknowledged that universities construct departments or faculties in diverse ways and can restructure over time, which can sometimes mean direct comparisons are less straightforward. Indeed, given the primary aim of this paper is to demonstrate the application of Propensity Score Matching (PSM) and Doubly Robust Estimation (DRE) in higher education evaluation research, future research could explore and incorporate a wider range of variables, including differences in student prior attainment, across departments and disciplines to provide a more nuanced understanding of the transition course’s impact on the outcomes of diverse student groups.

## Conclusion

This paper provided a worked example of how we can potentially make use of existing and available institutional data to generate more robust evidence to evaluate access and participation activities in higher education. We used the statistical methods of Propensity Score Matching (PSM) and Doubly Robust Estimation and found, from our initial analysis, a statistically significant positive difference between enrolment in a transition course and the likelihood of passing Year 1. Additional analysis that included the year before the course was available found no statistical difference in passing year 1 when compared to only those who enrolled in the course the following year, highlighting the different impacts in the short and longer terms. We hope to have provided sufficient details and insights into our decision-making processes and how statistical methods can be used with large institutional data, which for us is also an exploratory journey due to the scarcity of similar literature, especially in UK higher education evaluation.

## Data Availability

The dataset and codes used in this study are available in the researchers’ university data repository, accessible to users in accordance with repository guidelines: https://doi.org/10.17864/1947.001336 (Wong and Fletcher, 2024).

## Note

1. B represents the ‘unstandardised regression coefficient,’ which indicates the direction and magnitude of the relationship between a variable and the outcome. Exp(B) is the exponentiated B, or the odds ratio, which quantifies how the odds of the outcome change with each unit increase in the variable. For example, an Exp(B) greater than 1 indicates higher odds of passing Year 1, while values below 1 indicate lower odds. The 95% Confidence Interval (CI) represents the range of plausible values for the true odds ratio. A CI excluding 1 indicates statistical significance. For example, an odds ratio of 1.50 with a 95% CI of [1.20, 1.80] suggests that the odds of passing Year 1 are 50% higher for this predictor, with the true value likely between 20% and 80%.
